# Modernization of Traditional Oriental Medicine: New Dosage Forms and Medical Instruments

**DOI:** 10.1155/2018/6960125

**Published:** 2018-06-10

**Authors:** Gihyun Lee, Wonnam Kim, Woojin Kim, Hanbing Li

**Affiliations:** ^1^National Development Institute of Korean Medicine, Gyeongsan, Republic of Korea; ^2^Kyung Hee University, Seoul, Republic of Korea; ^3^Yale University, New Haven, CT, USA; ^4^Semyung University, Jecheon, Republic of Korea; ^5^Zhejiang University of Technology, Hangzhou, China

Traditional Oriental Medicine (TOM) has been developed for thousands of years; however recent developments in pharmaceutical technology promote modernization of TOM rapidly to expand accessibility, ease of administration, and cost-effectiveness. Various forms of herbal medicine are improved for better efficiency and consumer preferences including soft extracts, granules, intranasal administrations, eye drop, drop pills, injection, and capsules. In-depth researches on safety and efficacy of these formulations are ongoing. Another trend on modernization of TOM is an integrated application with technology. Progress on technology has developed oriental medical instruments. For example, modern electrical engineering makes electroacupuncture, laser acupuncture, and laser moxibustion possible. Experimental and translational studies on safety and efficacy of these new methods are underway.

Here we highlight some of the key ongoing challenges published in this special issue. S. Y. Suh and W. G. An used a modernized method to understand herbal medicine, such as herb-compound-target network and target-pathway network analysis, to study Bulsu-san commonly used for pregnant women in East Asia. In the results, the authors report that most compounds in Bulsu-san work together with multiple target genes in a synergetic way. C.-C. Yang et al. and M.-L. Lin et al. used new treatment methods. C.-C. Yang et al. investigated vascular and autonomic impacts of combined acupuncture-far infrared radiation in improving peripheral circulation. M.-L. Lin et al. evaluated the effectiveness of laser acupuncture plus Chinese cupping in chronic nonspecific lower back pain treatment, which could further be a suitable option for lower back pain treatment in clinical settings. Y.-S. Lee et al. and T. Shu et al. integrated technology to increase understanding of TOM. Y.-S. Lee et al. used a data mining procedure, determined by the application of a term frequency-inverse document frequency weighting scheme to the cooccurrence table, to analyze relationships between emotions and the visceral system according to the principles of East Asian medicine. T. Shu et al. proposed an effective noninvasive computerized method based on facial images to quantitatively detect heart disease.

In this special issue, we present 14 papers that address the issue about modernization of TOM, focusing on new approaches by herbal medicine and medical instruments.

*Gihyun Lee* *Wonnam Kim* *Woojin Kim* *Hanbing Li*

